# Supplementary material for: Modeling glioblastoma heterogeneity as a dynamic network of cell states
Source: Mol Syst Biol. 2021 Sep 16;17(9):e10105. doi: 10.15252/msb.202010105 (PMC8444284; doi:10.15252/msb.202010105)
Supplement: Supplementary file 6 — Source Data for Figure 5 [file MSB-17-e10105-s004.zip › Figure5A_sourcedata/GSEA_3071/hallmarks_stateVI.GseaPreranked.1623742557031/HALLMARK_BILE_ACID_METABOLISM.html]

Details for gene set HALLMARK\_BILE\_ACID\_METABOLISM[GSEA]

|  || Dataset | state63071 |
| Phenotype | NoPhenotypeAvailable |
| Upregulated in class | na\_neg |
| GeneSet | HALLMARK\_BILE\_ACID\_METABOLISM |
| Enrichment Score (ES) | -0.3754291 |
| Normalized Enrichment Score (NES) | -1.2360765 |
| Nominal p-value | 0.23994254 |
| FDR q-value | 0.45918787 |
| FWER p-Value | 1.0 |
Table: GSEA Results Summary

  

Fig 1: Enrichment plot: HALLMARK\_BILE\_ACID\_METABOLISM      
 Profile of the Running ES Score & Positions of GeneSet Members on the Rank Ordered List

  

| PROBE | GENE SYMBOL | GENE\_TITLE | RANK IN GENE LIST | RANK METRIC SCORE | RUNNING ES | CORE ENRICHMENT || 1 | DIO2 |  |  | 567 | 0.434 | -0.1018 | No |
| 2 | PEX16 |  |  | 1061 | 0.335 | -0.1956 | No |
| 3 | PEX6 |  |  | 1263 | 0.308 | -0.2123 | No |
| 4 | ACSL1 |  |  | 1605 | 0.268 | -0.2726 | No |
| 5 | AR |  |  | 1810 | -0.251 | -0.2974 | No |
| 6 | SLC23A2 |  |  | 2036 | -0.283 | -0.3238 | Yes |
| 7 | PNPLA8 |  |  | 2093 | -0.294 | -0.3022 | Yes |
| 8 | HSD17B4 |  |  | 2322 | -0.337 | -0.3227 | Yes |
| 9 | SLC35B2 |  |  | 2469 | -0.370 | -0.3164 | Yes |
| 10 | IDH1 |  |  | 2526 | -0.384 | -0.2835 | Yes |
| 11 | ATXN1 |  |  | 2860 | -0.481 | -0.3147 | Yes |
| 12 | IDH2 |  |  | 2966 | -0.524 | -0.2777 | Yes |
| 13 | PXMP2 |  |  | 3086 | -0.579 | -0.2376 | Yes |
| 14 | FADS2 |  |  | 3173 | -0.640 | -0.1808 | Yes |
| 15 | EFHC1 |  |  | 3259 | -0.719 | -0.1136 | Yes |
| 16 | RBP1 |  |  | 3264 | -0.723 | -0.0237 | Yes |
| 17 | SLC29A1 |  |  | 3449 | -1.005 | 0.0522 | Yes |
Table: GSEA details [plain text format]

  

Fig 2: HALLMARK\_BILE\_ACID\_METABOLISM: Random ES distribution      
 Gene set null distribution of ES for **HALLMARK\_BILE\_ACID\_METABOLISM**
